# Cardiac-specific catalase overexpression rescues anthrax lethal toxin-induced cardiac contractile dysfunction: role of oxidative stress and autophagy

**DOI:** 10.1186/1741-7015-10-134

**Published:** 2012-11-07

**Authors:** Machender R Kandadi, Xuejun Yu, Arthur E Frankel, Jun Ren

**Affiliations:** 1 Center for Cardiovascular Research and Alternative Medicine, University of Wyoming College of Health Sciences, Laramie, WY 82071, USA; 2 Cancer Research Institute of Scott & White Memorial Hospital, Temple, TX 76502, USA

**Keywords:** lethal toxin, cardiomyocyte, contractile function, autophagy, UPS

## Abstract

**Background:**

Lethal and edema toxins secreted by *Bacillus anthracis *during anthrax infection were found to incite serious cardiovascular complications. However, the underlying mechanisms in anthrax lethal toxin-induced cardiac anomalies remain unknown. This study was designed to evaluate the impact of antioxidant enzyme catalase in anthrax lethal toxin-induced cardiomyocyte contractile dysfunction.

**Methods:**

Wild type (WT) and cardiac-specific catalase overexpression mice were challenged with lethal toxin (2 μg/g, intraperotineally (i.p.)). Cardiomyocyte contractile and intracellular Ca^2+ ^properties were assessed 18 h later using an IonOptix edge-detection system. Proteasome function was assessed using chymotrypsin-like and caspase-like activities. GFP-LC3 puncta and Western blot analysis were used to evaluate autophagy and protein ubiquitination.

**Results:**

Lethal toxin exposure suppressed cardiomyocyte contractile function (suppressed peak shortening, maximal velocity of shortening/re-lengthening, prolonged duration of shortening/re-lengthening, and impaired intracellular Ca^2+ ^handling), the effects of which were alleviated by catalase. In addition, lethal toxin triggered autophagy, mitochondrial and ubiquitin-proteasome defects, the effects of which were mitigated by catalase. Pretreatment of cardiomyocytes from catalase mice with the autophagy inducer rapamycin significantly attenuated or ablated catalase-offered protection against lethal toxin-induced cardiomyocyte dysfunction. On the other hand, the autophagy inhibitor 3-MA ablated or significantly attenuated lethal toxin-induced cardiomyocyte contractile anomalies.

**Conclusions:**

Our results suggest that catalase is protective against anthrax lethal toxin-induced cardiomyocyte contractile and intracellular Ca^2+ ^anomalies, possibly through regulation of autophagy and mitochondrial function.

## Background

The 2001 anthrax bioterrorism in the United States has drawn the interest of the scientific community in understanding the pathophysiology of anthrax infection. Anthrax is a pathological condition caused by a spore-forming, Gram-positive bacterium *Bacillus anthracis*. Infection by inhalation of *B. anthracis *spores can result in a mortality rate up to 96% [1-3]. Major routes of infection have been confirmed through inhalation of, skin contact with or ingestion of *Bacillus anthracis *spores. Anthrax toxin is the major virulence factor of *Bacillus anthracis*, containing three polypeptides, namely: edema factor (EF), lethal factor (LF) and protective antigen (PA). LF is a zinc metalloprotease which specifically cleaves the NH_2_-terminal of mitogen-activated protein kinase kinases resulting in inactivation of the kinases. EF is a calmodulin-dependent adenylyl cyclase which promotes intracellular cAMP accumulation and associated cellular responses [4-7]. PA binds the cellular receptors tumor endothelial marker 8 and capillary morphogenesis protein 2 [8,9]. The combination of LF and the receptor binding PA yields the lethal toxin [10]. Once bound to the receptor and proteolytically activated, PA forms a heptamer to deliver EF and/or LF to the cytoplasm following receptor-mediated endocytosis. Following anthrax exposure, patients usually develop refractory hypotension unresponsive to antibiotics, fluid, pressor and respiratory support [11]. Anthrax lethal toxin was found to decrease the heart rate, left ventricular ejection fraction and mean arterial pressure [12,13]. In addition, anthrax lethal toxin has been reported to directly compromise myocardial function [14-17]. However, the underlying mechanisms behind lethal toxin-induced unfavorable cardiac effects remain elusive.

Accumulation of reactive oxygen species (ROS) has been known to trigger cellular injury, including oxidation of DNA and lipids, mitochondrial damage and dysregulated autophagy [18,19]. Evidence suggests that anthrax lethal toxin initiates ROS accumulation; in particular, generation of superoxide and other ROS in macrophages and neutrophils [14,20,21]. We previously reported that anthrax lethal toxin stimulates myocardial superoxide generation and thus impairs cardiac contractility [14]. To this end, our present study was designed to examine the effect of the antioxidant enzyme catalase on lethal toxin-induced cardiac contractile anomalies and the underlying mechanism. Catalase is an antioxidant enzyme converting hydrogen peroxide (H_2_O_2_) produced from highly reactive superoxide (O_2_^-^) by superoxide dismutase to water and oxygen molecules. Given that autophagy has been implicated in anthrax infection [14], essential protein markers for autophagy, including microtubule- associated protein light chain 3 (LC3), Beclin-1, autophagy related gene-7 (Atg-7) and green fluorescent protein-tagged LC3 puncta (GFP-LC3), were monitored in myocardial tissues or H9C2 myoblasts with or without lethal toxin challenge. Given the pivotal role of ubiquitin-proteasome system (UPS) in maintaining the protein synthesis and degradation parallel to the autophagic quality control mechanism [22], proteasome function was assessed using chymotrypsin-like and caspase-like activities.

## Methods

### Generation of catalase overexpression transgenic mice and production of anthrax lethal toxin

Catalase overexpressing transgenic mouse generation was described in detail previously [23]. In brief, an 8-kb CAT driven by α-myosin heavy chain (α-MHC) promoter containing the entire coding sequences of the catalase cDNA was purified on a matrix of diatomaceous earth (Prepagene, Bio-Rad Hercules, CA, USA) and filtered through a 0.22-μm filter. Approximately, 100 copies of the purified transgene insert were microinjected into one pronucleus of each one-cell mouse embryo of the inbred strain FVB. The transgene transcription of catalase was controlled by the mouse α-MHC gene. To identify transgenic founder mice, genomic DNA was isolated from 1-cm tail clips from four-week-old mice. DNA was subjected to Southern and dot blot analyses, which were probed with a 550-bp *Sma*I/*Not*I fragment derived from the rat insulin II portion of the CAT. This probe hybridized to an 8,000-bp*Eco*RI fragment of the transgene, consistent with the presence of a unique *Eco*RI site in the CAT (600-bp upstream of the MHC transcription initiation site). Founder mice were bred with mice of the same strain and transgenic offspring were routinely identified by a polymerase chain reaction (PCR) using a primer pair derived from the MHC promoter and rat catalase cDNA with the reverse sequence of AAT ATC GTG GGT GAC CTC AA and the forward sequence of CAG ATG AAG CAG TGG AAG GA. These transgenic mice have approximately 60-fold catalase overexpression.

Recombinant PA and LF were produced and purified as previously described [15,24,25]. In brief, plasmid pSJ115 encoding LF from SCS110 *E. coli *stocks was transformed into BH445 crippled *B. anthracis *strain by electroporation [24]. Single colonies from LB kanamycin agar plates were grown overnight and 20 mL culture media were inoculated into 6 L modified FA media in the presence of 1 mL polypropylene glycol P200 and kanamycin at 37^°^C at 100 to 300 rpm agitation rate and 2 L air/min constant sparging for 14 h. Cultures were centrifuged, and 2 mM EDTA and 0.1 mM PMSF added to supernatant. Supernatants were sterilized using 0.22-μm filters and concentrated to 300 mL with 10,000 Da Mr cutoff cellulose membranes in a Pellicon system. A total of 40% ammonium sulfate was added, and the sample was loaded, washed and eluted from Fast Flow PhenylSepharose six columns with a 1.5 to 0 M ammonium sulfate gradient in 20 mM Hepes/5 mM EDTA pH 7.5. LF containing fractions were concentrated in Centricon Plus-80, dialyzed into 20 mM Hepes/0.1 mM EDTA pH 8, and eluted from a Q-Sepharose Fast Flow column with a 0 to 0.5 M NaCl in 10 mM Tris/0.5 mM EDTA pH 8 gradient. Eluted LF fractions were pooled, concentrated again with Centricon Plus-80 filters, diluted in 1x PBS, filter sterilized and frozen at -80^°^C. pYS5-wild-type PA vector was transformed into *B. anthracis *as described above. Colonies were expanded in overnight cultures with kanamycin and were inoculated into 6 L modified FA medium with polypropylene glycol and kanamycin for LF and PA fermentation similar to LF [25]. After 14 h, cultures were centrifuged prior to the addition of EDTA and PMSF. Filtration and concentration were conducted using a Pellicon system. A total of 20% ammonium sulfate was added, and PA loaded, washed and eluted from Phenyl Sepharose Fast Flow with a 1.5 to 0 M ammonium sulfate gradient in 10 mM HEPES/5 mM EDTA pH 7. PA containing fractions were dialyzed against 20 mM Tris/5 mM EDTA pH 8.9 and loaded and eluted from a Q Sepharose Fast Flow column with a 0 to 0.5 M NaCl in 20 mM Tris/5 mM EDTA pH 8.9 gradient. Pooled PA fractions were purified using the Superdex 75 chromatography in PBS/5 mM EDTA pH 7.4 and were pooled, concentrated with Centricon Plus-80 filters, diluted in 1x PBS, filter sterilized and frozen at -80^°^C until experimentation. Immediately before *in vivo *injection, toxin components were thawed and mixed in PBS.

### Experimental animals and anthrax lethal toxin challenge

All animal procedures used in this study were approved by the Animal Care and Use Committee at the University of Wyoming (Laramie, WY, USA). Animals were housed under well-controlled conditions of temperature (22 ± 2^°^C), humidity (55 ± 5%) and circadian cycle (12 h/12 h light-dark) with access to food and water *ad libitum*. Four month-old adult male cardiac-specific catalase overexpressing (CAT) mice and their wild-type (WT) littermates were used. For lethal toxin challenge, mice were injected intraperitoneally with PBS or anthrax lethal toxin (2 μg/g) (LF + PA 1:2 ratio) and were sacrificed 18 h following *in vivo *lethal toxin injection [14].

### Murine cardiomyocyte isolation and drug treatments

After ketamine/xylazine (80 mg/kg:12 mg/kg, i.p.) sedation, hearts were removed and perfused with Ca2+ -free Tyrode's solution containing (in mM): NaCl 135, KCl 4.0, MgCl_2 _1.0, HEPES 10, NaH_2_PO_4 _0.33, glucose 10, butanedione monoxime 10 and the solution was gassed with 5% CO_2_/95% O_2_. Hearts were digested with Liberase Blendzyme 4 (Hoffmann-La Roche Inc., Indianapolis, IN, USA) for 20 minutes. Left ventricles were removed and minced before being filtered. Extracellular Ca^2+ ^was added incrementally back to 1.20 mM over a period of 30 minutes. Myocyte yield was approximately 75%, which was not affected by either lethal toxin challenge or catalase overexpression. Only rod-shaped myocytes with clear edges were selected for mechanical study [14]. To assess the cause-effect relationship of autophagy in lethal toxin-induced cardiomyocyte anomalies, *in vitro *studies were performed using cardiomyocytes isolated from WT and catalase transgenic mice treated with or without lethal toxin (100 ng/ml) at 37^°^C for 3 h in the presence or absence of the autophagy inhibitor 3-methyladenine (3-MA, 10 mM) or the autophagy inducer rapamycin (5 μM) prior to mechanical and biochemical evaluation [26].

### Cell shortening and relengthening

Mechanical properties of cardiomyocytes were evaluated using a MyoCam system (IonOptix Corporation, Milton, MA, USA) [14]. Briefly, cardiomyocytes were visualized under an inverted microscope (IX-70, Olympus Optical Co., Tokyo, Japan) and were stimulated at a frequency of 0.5 Hz. The myocyte being observed was displayed on a computer monitor using an IonOptix MyoCam camera (IonOptix Corporation, Milton, MA, USA). IonOptix SoftEdge software was utilized to capture cell shortening and re-lengthening. The indices measured included peak shortening (PS), time-to-peak shortening (TPS), time-to-90% re-lengthening (TR_90_), and maximal velocity of shortening/re-lengthening (± dL/dt).

### Intracellular Ca^2+ ^transients

Isolated cardiomyocytes were loaded with fura-2/AM (0.5 μM) for 10 minutes. Fluorescence intensity was recorded with a dual-excitation fluorescence photomultiplier system (IonOptix). Myocytes were placed onto an Olympus IX-70 inverted microscope and imaged through a Fluor 40x objective. Cells were exposed to light emitted by a 75 W lamp and passed through either a 360 or a 380 nm filter while being stimulated to contract at 0.5 Hz. Fluorescence emissions were detected between 480 and 520 nm, and qualitative change in fura-2 fluorescence intensity was inferred from the fura-2 fluorescence intensity ratio at 360 and 380 nm. Fluorescence decay time (both single and bi-exponential curve fits) was calculated as an indicator of intracellular Ca^2+ ^clearing [14].

### Measurement of O_2_^- ^levels in myocardium

*In situ *production of myocardial O_2_^- ^was assessed using the oxidative fluorescent dye dihydroethidium (DHE) [14]. The cell membrane permeable DHE is oxidized to fluorescent hydroxyethidine by O_2_^- ^and then intercalated into DNA. In brief, myocardial tissues were placed in ice-cold PBS, rinsed and embedded in the optimal cutting temperature (OCT) compound medium (Sakura Finetek USA, Inc., Torrance, CA, USA) for cryosectioning. The cryomoulds were stored at -80^°^C. Sections (30 μm) from the cryomoulds were thawed to room temperature and were incubated with DHE (10 μM) for 45 minutes at room temperature in a dark chamber. After being washed three times with PBS, sections were fixed with aqueous mounting medium (VectaMount AQ, Vector Laboratories, Burlingame, CA, USA) and images were obtained using a Zeiss LSM 710 confocal microscope (Carl Zeiss MicroImaging GmbH, Jena, Germany). Nuclei with red fluorescence were measured using the ImageJ analysis software (version 1.34S, developed by NIH).

### Measurement of ROS production

Production of cellular reactive oxygen species (ROS) was evaluated by analyzing the changes in fluorescence intensity resulting from oxidation of the intracellular fluoroprobe 5-(6)-chloromethyl-2, 7-dichlorodihydrofluorescein diacetate (CM-H2DCFDA). In brief, cardiomyocytes were isolated from WT and catalase transgenic mice challenged with or without lethal toxin (2 μg/g, i.p., for 18 h) and were loaded with 10 μM of the nonfluorescent dye CM-H2DCFDA (Molecular Probes, Eugene, OR, USA) at 37°C for 30 minutes. The myocytes were rinsed with KRH buffer (125 mM NaCl, 5 mM KCl, 1.8 mM CaCl_2_, 2.6 mM MgSO_4_, 5 mM HEPES, pH 7.4) and the fluorescence intensity was then measured with a SpectraMax XS fluorescence microplate spectrophotometer (Spectra MaxGeminiXS, spectra Max, Atlanta, GA, USA) at an excitation wavelength of 480 nm and an emission wavelength of 530 nm. Sections (30 μm) of frozen tissues were thawed to room temperature and were incubated with CM-H_2_DCFDA (10 μM) for 1 h at room temperature in a dark chamber. After being washed three times with PBS, sections were fixed with aqueous mounting medium (VectaMount AQ, Vector Laboratories) and images were obtained using a Zeiss LSM 710 confocal microscope (Carl Zeiss MicroImaging GmbH, Jena, Germany) [27].

### Cell culture

H9C2 cells, a clonal cell line derived from fetal rat heart, purchased from ATCC (Manassas, VA, USA). Cells were grown in Dulbecco's modified Eagle's medium (DMEM) supplemented with 10% fetal bovine serum (FBS) (Gibco, Grand Island, NY, USA) and 1% penicillin and streptomycin and maintained in 95% air and 5% CO_2 _at 37^°^C. Cells were grown to 80% confluence before treating with anthrax lethal-toxin [14].

### Measurement of mitochondrial membrane potential (ΔΨm)

Murine cardiomyocytes from lethal toxin-treated mice or H9C2 cells following *in vitro *lethal-toxin treatment (100 ng/ml for 1 h) were incubated with JC-1 (5 μM) for 15 minutes at 37^°^C. Fluorescence was analyzed with a Texas red-FITC filter cube (Spectra MaxGeminiXS, spectra Max, Atlanta, GA, USA). Red emission represents a potential-dependent aggregation in the mitochondria, reflecting ΔΨm. Green fluorescence represented the monomeric form of JC-1, appearing in the cytosol after mitochondrial membrane depolarization. Fluorescence of each sample was read at an excitation wavelength of 490 nm and emission wavelength of 530 (Green) and 590 (Red) nm using a spectrofluorimeter (Spectra MaxGeminiXS, spectra Max, Atlanta, GA, USA) at an interval of 10 sec. Results in fluorescence intensity were expressed as 590-to-530 nm emission ratio. The mitochondrial uncoupler carbonyl cyanide m-cholorophenylhydrazone (CCCP, 50 μmol/L) was used as a positive control for ΔΨm measurement [28].

### Proteasome activity

Proteasome activity was measured as described [29]. Briefly, heart tissues were homogenized in HEPES buffer (in mM: NaCl 137, KCl 4.6, KH_2_PO_4 _1.1, MgSO_4 _0.6, EDTA 1, and DTT 1) without protease inhibitor at 4^°^C and then centrifuged at 16,168 g to obtain the soluble fraction. Protein (50 μg in 50 μl) was incubated in 50 μl of 50 mM Tris-HCl buffer, pH 7.5, containing 20 mM KCl, 0.5 mM MgCl_2 _and 1 mM DTT for 1 h with 200 μM fluorogenic substrates Suc-LLVY-AMC/Z-LLE-AMC (Enzo Life Sciences, Plymouth Meeting, PA, USA). Fluorescence products were measured using a spectrofluorimeter (Spectra MaxGeminiXS, spectra Max, Atlanta, GA, USA) using a 355-nm excitation and 460-nm emission filter.

### LC3B-GFP-adenovirus production and infection

Adenovirus containing LC3-GFP construct (GFP tagged on the N-terminal of LC3) was kindly provided by Dr. Cindy Miranti (Van Andel Institute, Grand Rapids, MI, USA) and was propagated using HEK293 cell line. Briefly, cells were infected with LC3-GFP adenovirus and collected upon plaque formation. Cell debris was collected by centrifugation, and aliquots of supernatant with viral particles were stored at -80°C. Adenovirus was purified using an Adeno-X Maxi purification kit from Clontech (Clontech Laboratories, Inc., Mountain View, CA, USA). H9C2 cells were grown to confluence on Lab-Tek chamber slides. Cells were then infected at an MOI of 2 with adenoviruses expressing LC3-GFP fusion protein. Medium was replaced with fresh DMEM after 6 h. Twenty four hours later, cells were observed for autophagy using confocal microscopy [14].

### Quantification of GFP-LC3

H9C2 cells transfected with GFP-LC3 adenovirus were treated with or without anthrax lethal toxin (100 ng/ml). Cells were fixed with 4% paraformaldehyde in PBS for 20 minutes at room temperature. Cells were then washed with PBS three times. These fixed cells were treated with DAPI for five minutes followed by three washes with PBS. Cover slips were mounted on slides using Vecta mount™ AQ-aqueous mounting medium (Vector Laboratories, Inc.,). For analysis of autophagy, cells were visualized at 40x magnification using a Zeiss LSM 710 confocal microscope (Carl Zeiss MicroImaging GmbH, Jena, Germany) and the percentage of cells showing numerous GFP-LC3 puncta (>10 dots/cells) were scored as described previously [30]. A minimum of 75 to 100 cells were scored for each condition in at least three independent experiments.

### Western blot analysis

Protein samples for Western blot analysis were prepared as described [14]. Briefly, ventricular tissues or H9C2 cells were homogenized and sonicated in a lysis buffer containing 20 mM Tris (pH 7.4), 150 mM NaCl, 1 mM ethylenediaminetetraacetic acid (EDTA), 1 mM ethyleneglycoltetraacetic acid (EGTA), 1% Triton, 0.1% SDS and 1% protease inhibitor cocktail. Equal amounts (50 μg) of proteins were separated on 7% to 15% SDS-polyacrylamide gels in a minigel apparatus (Mini-PROTEAN II, Bio-Rad Laboratories Inc.,) and were transferred electrophoretically to nitrocellulose membranes. The membranes were blocked with 5% milk in Tris-buffered saline before overnight incubation at 4°C with anti-Beclin-1 (1:1,000), anti-Atg-7 (1:1,000), anti-LC3-II (1:1,000), anti-phospholamban (1:1,000), anti-phosphorylated phospholamban (1:1,000), anti-SERCA2a (1:1,000), anti-Na^+^-Ca^2+ ^exchanger (NCX, 1:1,000), anti-ubiquitin (1:1000), and anti-GAPDH (loading control, 1:1,000) antibodies. Proteins were visualized after subsequent incubation with a 1:5,000 dilution of anti-mouse or anti-rabbit IgG conjugated to horseradish peroxidase and a LumiGLO^® ^Chemiluminescence detection procedure (Cell Signaling Technologies, Beverly, MA, USA).

### Statistical analysis

Data were expressed as mean ± SEM. Statistical significance (*P *<0.05) for each variable was estimated by analysis of variance (ANOVA) followed by the Tukey's test for *post-hoc *analysis.

## Results

### Effect of lethal toxin challenge on survival rate and cardiomyocyte mechanics in FVB and catalase transgenic mice

**Figure 1:**
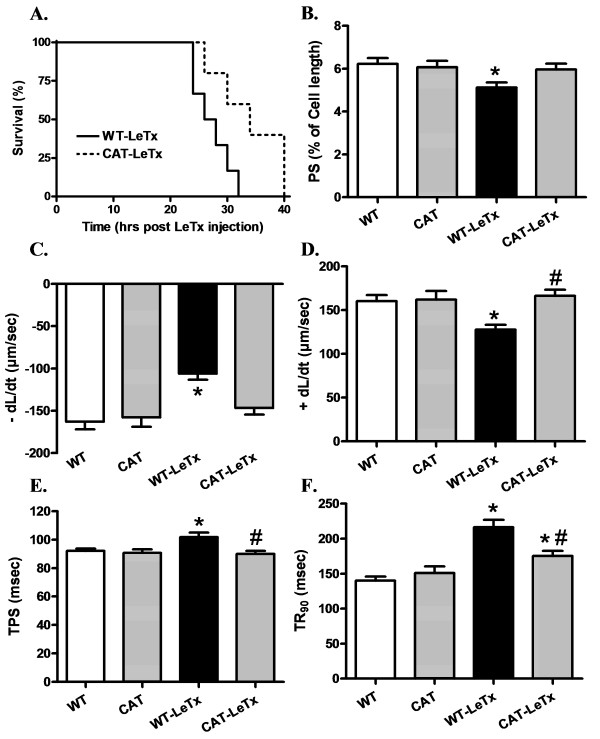
**Effect of LeTx on survival rate and cell shortening in cardiomyocytes from WT and catalase mice**. **A**: Survival rate (%); **B**: Peak shortening (PS, normalized to resting cell length); **C**: Maximum velocity of shortening (+ dL/dt); **D**: Maximum velocity of relengthening (-dL/dt); **E**: time-to-peak shortening (TPS); and **F**: time-to-90% re-lengthening (TR_90_). For panel A; n = 8 mice per group. For panel B-F data were presented as Mean ± SEM, n = 100 to 150 cells from two to three mice per group, *P <0.05 *vs*. WT group, # *P *<0.05 vs. WT-LeTx group.

**Figure 2:**
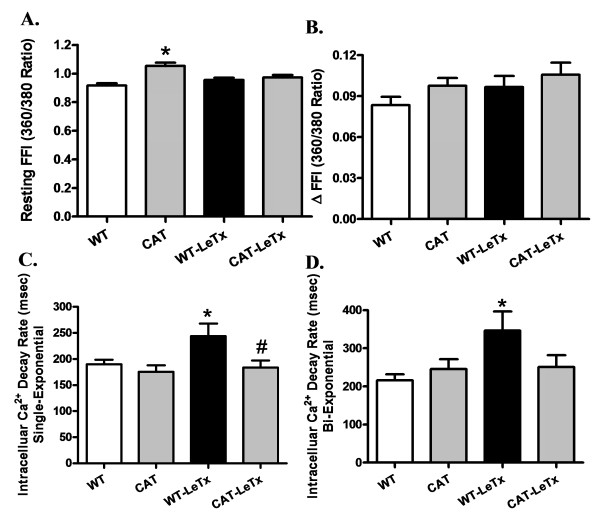
**Effect of LeTx on intracellular Ca^2+ ^transients in cardiomyocytes from WT and catalase mice**. **A**: Resting fura-2 fluorescence intensity (FFI); **B**: electrically stimulated rise in FFI (ΔFFI); **C**: Single exponential intracellular Ca^2+ ^transient decay rate; and **D**: Bi-exponential intracellular Ca^2+ ^transient decay rate. Mean ± SEM, n = 60 to 75 cells from two to three mice per group, *P <0.05 *vs*. WT group, #*P *<0.05 *vs*. WT-LeTx group.

The Kaplan-Meier survival curve depicts that catalase transgenic mice survived approximately 8 h longer than WT mice although there was 100% mortality ultimately in WT and catalase transgenic mice following lethal toxin treatment (Figure 1A). Lethal toxin challenge significantly reduced peak shortening (PS) and maximal velocity of shortening/re-lengthening (±dL/dt) as well as prolonged time-to-PS (TPS) and time-to-90% re-lengthening (TR_90_) in WT cardiomyocytes, the effect of which was either significantly attenuated or ablated by catalase overexpression. Catalase overexpression itself failed to affect the cardiomyocyte mechanics tested (Figure 1B-F). To explore the potential mechanisms of action behind lethal toxin-induced cardiomyocyte mechanical abnormalities, intracellular Ca^2+ ^handling was evaluated using fura-2 fluorescence in cardiomyocytes from WT and catalase transgenic mice with or without lethal toxin exposure. Cardiomyocytes from catalase transgenic mice displayed subtle but significant higher basal intracellular Ca^2+ ^levels although none of the other intracellular Ca^2+ ^indices was altered by catalase overexpression. Lethal toxin exposure significantly prolonged intracellular Ca^2+ ^decay rate (both single and bi-exponential curve fit) without affecting the resting and electrically stimulated rise in intracellular Ca^2+ ^levels, the effects of challenge were significantly attenuated or ablated by catalase overexpression (Figure 2).

### Effect of lethal toxin and catalase overexpression on intracellular Ca^2+ ^regulatory proteins

To further determine the mechanism underscoring lethal toxin exposure- and catalase overexpression-elicited changes in intracellular Ca^2+ ^homeostasis, levels of intracellular Ca^2+ ^regulatory proteins including SERCA2a, Na^+^-Ca^2+ ^exchanger and the SERCA inhibitory protein phospholamban were examined. Our data shown in Additional file 1, Figure S1 revealed that neither lethal toxin nor catalase, or both, affected levels of SERCA2a, Na^+^-Ca^2+ ^exchanger and phospholamban (or its phosphorylation).

### Effect of catalase overexpression on lethal toxin exposure-induced O_2_^- ^and ROS production

**Figure 3:**
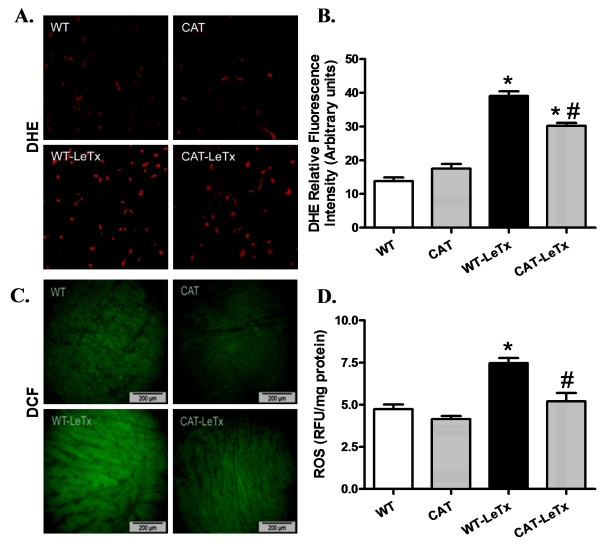
**Effect of catalase (CAT) overexpression on LeTx-induced generation of O_2_^- ^and ROS**. **A**: Representative images depicting DHE fluorescence in myocardium from WT and catalase transgenic mice treated with or without lethal toxin. **B**: Pooled data of O_2_^- ^production; **C**: Representative images depicting DCF fluorescence in myocardium from WT and catalase transgenic mice; and D: ROS levels in isolated cardiomyocytes from WT and catalase transgenic mice treated with or without lethal toxin. Mean ± SEM, n = 3 mice (panel B) or 6 isolations (panel D) per group, *P <0.05 *vs*. WT group, #*P *<0.05 *vs*. WT-LeTx group.

O_2_^- ^production was significantly elevated in myocardium from lethal toxin-treated WT mice, the effect of which was significantly attenuated by catalase overexpression with little effect of catalase by itself (Figure 3A-B). In addition, our data depicted that lethal toxin significantly elevated ROS generation in WT cardiomyocytes, the effect of which was mitigated by catalase overexpression. Catalase overexpression itself did not affect ROS production (Figure 3C-D).

### Effect of catalase overexpression on lethal toxin exposure-induced mitochondrial dysfunction

**Figure 4:**
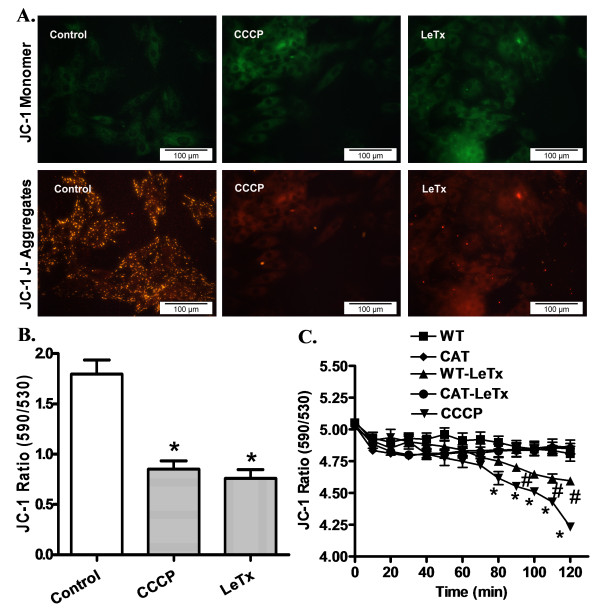
**Effect of catalase (CAT) overexpression on LeTx-induced cardiomyocyte mitochondrial damage**. **A**: Representative fluorescent images of JC-1 in control and LeTx exposure conditions. CCCP (10 μM) was used as a positive control; **B**: Summarized JC-1 ratio representing mitochondrial membrane potential in response to LeTx and CCCP exposure; and **C**: Cardiomyocyte mitochondrial membrane potential over time. Mean ± SEM, n = 6 isolations per group, *P <0.05 *vs*. WT group, #*P *<0.05 *vs*. WT-LeTx group.

Mitochondrial membrane potential (ΔΨm) is well known as an essential indicator for mitochondrial and ultimately cardiomyocyte viability and function [31]. The cationic lipophilic probe JC-1 was employed to monitor ΔΨm in response to lethal toxin treatment. The dynamic change of ΔΨm was displayed by changes in the red (aggregated JC-1) and green (monomeric form of JC-1) fluorescence. Quantitative analysis exhibited a significant reduction in the ratio between the red and green fluorescence in response to lethal toxin treatment, indicating a fall in ΔΨm and mitochondrial damage. Interestingly, the lethal toxin-induced fall in ΔΨm was abrogated by catalase overexpression. Catalase overexpression itself did not exert any significant effect on ΔΨm (Figure 4).

### Effect of catalase overexpression on lethal toxin-induced ubiquitin-proteasome dysfunction

**Figure 5:**
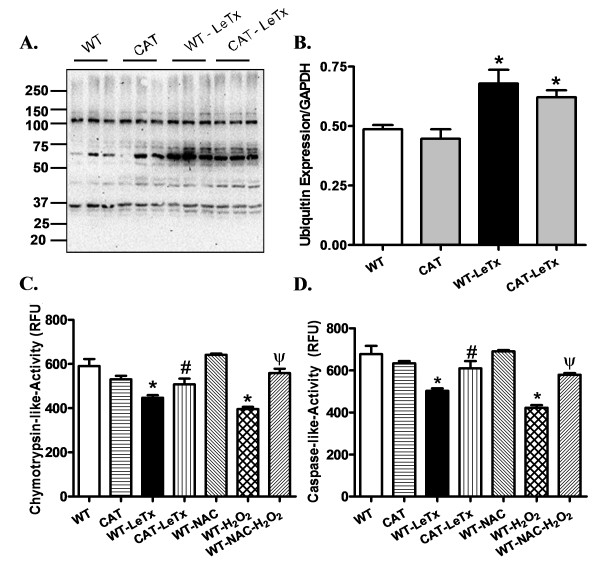
**Effect of catalase (CAT) overexpression and antioxidant on LeTx-induced changes in ubiquitin-proteasome activity**. Expression and activity of the ubiquitin-proteasome system were determined in myocardium from WT and catalase transgenic mice treated with or without lethal toxin. For positive control, a cohort of isolated cardiomyocytes from WT mice were incubated with H_2_O_2 _(250 μM) for 2 h in the presence or absence of the antioxidant NAC (500 μM). **A**: Representative gel blots in triplicates depicting ubiquitination; **B**: Ubiquitin expression; **C**: Chymotrypsin-like activity; and **D**: Caspase-like activity. Mean ± SEM, n = 6 mice or isolations per group, *P <0.05 *vs*. WT group, #*P *<0.05 *vs*. WT-LeTx group, ψ *P *<0.05 *vs*. WT-H_2_O_2 _group.

Protein degradation by the ubiquitin-proteasome system involves attachment of multiple ubiquitin molecules to substrate protein followed by degradation of the tagged protein by the 26S proteasome complex. Basal ubiquitination in hearts from WT and catalase transgenic mice was comparable. Interestingly, lethal toxin exposure significantly up-regulated ubiquitination while it down-regulated proteasome activity as measured by chymotrypisn-like and caspase-like activities in WT mice. Catalase overexpression abrogated lethal toxin-induced increase in ubiquitination and decrease in chymotrypsin-like and caspase-like activities. Similarly, isolated cardiomyocytes treated with H_2_O_2 _(250 μM) for 2 h elicited a significant inhibition of both chymotrypsin- and caspase-like activities, the effect of which was ablated by the pretreatment with the antioxidant N-acetyl-cysteine (NAC, 500 μM) (Figure 5).

### Effect of anthrax lethal toxin on autophagosome formation

**Figure 6:**
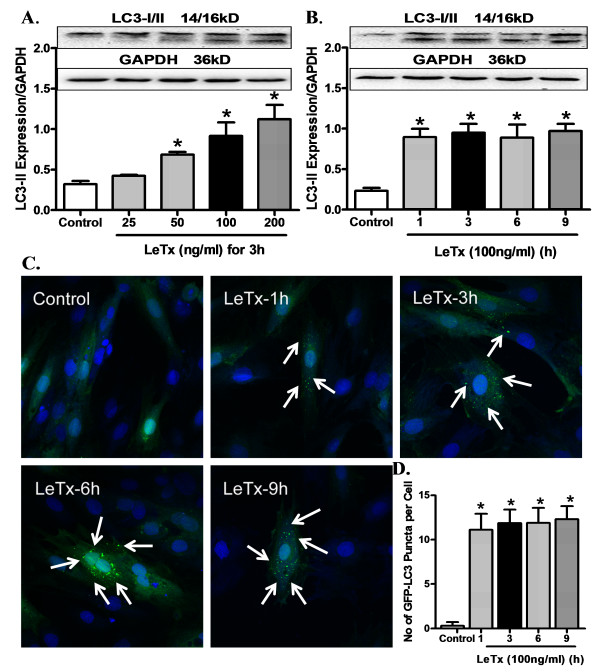
**Effect of LeTx on autophagosome formation**. **A**: LC3-II in H9C2 cells treated with different concentrations of lethal toxin (25 to 200 ng/ml); **B**: LC3-II in H9C2 cells treated with 100 ng/ml lethal toxin for different durations of times (1 to 9 h). Inserts depicting expression LC3-I/II and GAPDH (loading control); **C**: Representative fluorescent microscopic images of GFP-LC3-II (DAPI's blue staining depicts nucleus; white arrows points autophagosomes); **D**: Quantitation of number of autophagosome per cell. Mean ± SEM, n = 3 independent cultures, *P <0.05 *vs*. Control.

To evaluate autophagosome formation in response to lethal toxin exposure, GFP tagged onto the N-terminal of LC3 (GFP-LC3) was used as a surrogate marker in H9C2 cells. With activation of autophagy, GFP-LC3-I is processed to GFP-LC3-II before getting recruited onto the autophagosome membrane (shown as punctuate using fluorescence microscopy). Our data revealed that lethal toxin treatment (25 to 200 ng/ml for 3 h) triggered significant up-regulation of LC3-II levels in a concentration dependent manner (Figure 6A). Lethal toxin (100 ng/ml) caused a significant up-regulation of autophagosome formation and LC3-II expression levels (Figure 6B-D). These results clearly indicated that lethal toxin induced overt autophagy.

### Effect of catalase overexpression on lethal toxin exposure-induced autophagy

**Figure 7:**
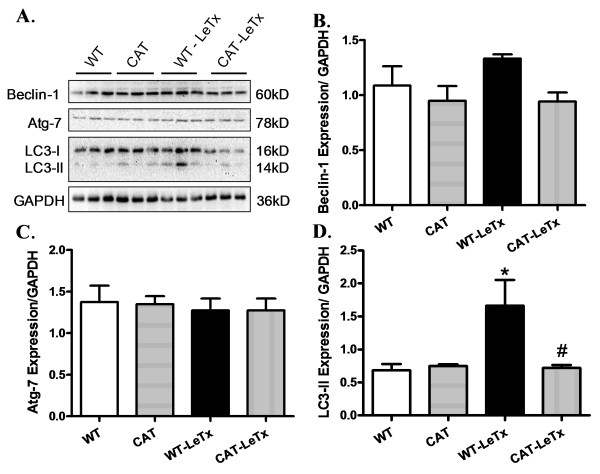
**Effect of catalase (CAT) overexpression on LeTx-induced autophagy**. **A**: Representative gel blot depicting expression of Beclin-1, Atg-7, LC3-I/II and GAPDH (loading control); Triplicates in the gel blot represents samples from three mice of each treatment group. **B**: Beclin-1; **C**: Atg-7; and **D**: LC3-II. Mean ± SEM, n = 6 mice per group, *P <0.05 *vs*. WT group, #*P *<0.05 *vs*. WT-LeTx group.

Overt autophagy has been implicated in cardiovascular complications [18]. LC3 exists in two forms, a 16-kDa cytosolic form (LC3-I) and a 14-kDa processed form (LC3-II) localized on autophagosome membrane [32]. Our data revealed elevated LC3-II levels without significant changes in the levels of Beclin-1 and Atg-7 in WT myocardium following lethal toxin exposure. Catalase transgenic overexpression itself did not significantly alter the expression of autophagic protein markers. However, catalase significantly attenuated lethal toxin-induced increase in LC3-II levels (Figure 7).

### Effect of ROS inhibition on lethal toxin-induced changes in autophagy proteins

**Figure 8:**
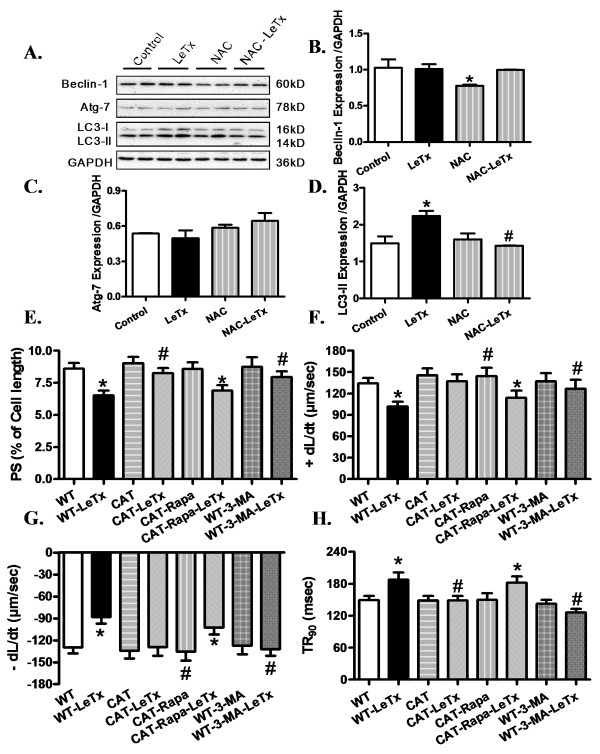
**Effect of ROS inhibition on lethal toxin-induced autophagy**. **A-D**: Isolated cardiomyocytes were incubated with LeTx (100 ng/ml) for 3 h in the absence or presence of the NAC (500 μM). A: Representative gel blots depicting expression of Beclin-1, Atg-7, LC-3 and GAPDH (used as loading control); B: Beclin-1; C: Atg-7; D: LC3-II. Each lane in a treatment group on the gel represents sample from independent experiment. Mean ± SEM, n = 4 independent isolations per group, *P <0.05 *vs*. control group, # *P *<0.05 vs. LeTx group. Effect of autophagy induction or inhibition on lethal toxin induced cardiac contractile dysfunction. **E-J**: Isolated cardiomyocytes from WT and CAT mice were incubated with lethal toxin (100 ng/ml) for 3 h in presence or absence of the autophagy inhibitor 3-methyladenine (3-MA, 10 mM) or autophagy inducer rapamycin (5 μM) respectively. E: Peak shortening (PS, normalized to resting cell length); F: Maximum velocity of shortening (+ dL/dt); G: Maximum velocity of relengthening (- dL/dt); H: time-to-90% re-lengthening (TR_90_). Mean ± SEM, n = 60 to 75 cells from three mice per group, *P <0.05 *vs*. WT group, # *P *<0.05 *vs*. WT-LeTx group.

To further consolidate the role of ROS in lethal toxin-induced autophagy, levels of Beclin-1, Atg-7 and LC3-II were determined. Lethal toxin exposure elicited significant up-regulation of LC3-II expression without affecting Beclin-1 and Atg-7. Interestingly, pretreatment with antioxidant NAC obliterated the lethal toxin-induced rise in LC3-II. NAC itself did not affect the expression of autophagy markers with the exception of a subtle, although significant, reduction in Beclin-1 level. These data favor a role of ROS in lethal toxin-induced autophagosome formation (Figure 8A-D).

### Effect of lethal toxin, 3-MA and rapamycin on cardiomyocyte contractile function

To further examine the causal role of autophagy in lethal toxin-induced contractile response, cardiomyocytes from WT and catalase transgenic mice were treated with or without lethal toxin in the absence or presence of the autophagy inducer rapamycin (5 μM) or the autophagy inhibitor 3-MA (10 mM). Our data showed that lethal toxin significantly depressed PS, ± dL/dt and prolonged TR_90 _without affecting resting cell length and TPS in WT cardiomyocytes, the effects of which were ablated or significantly attenuated by catalase overexpression. Interestingly, pretreatment of cardiomyocytes from catalase transgenic mice with rapamycin prior to lethal toxin exposure significantly attenuated or ablated catalase-offered protection against a lethal toxin-induced defect. To the contrary, the autophagy inhibitor 3-MA effectively ablated or significantly attenuated lethal toxin-induced cardiomyocyte mechanical abnormalities (Figure 8E-H, Additional file 1 Figure S2). These data revealed a permissive role of autophagy in lethal toxin-induced cardiomyocyte contractile anomalies.

## Discussion

The salient findings of our study are that anthrax lethal toxin exposure elicits enhanced ROS accumulation, myocardial contractile dysfunction, impaired intracellular Ca^2+ ^handling, decreased mitochondrial membrane potential, enhanced ubiquitination and overt autophagy. Intriguingly, cardiac catalase overexpression abrogated or attenuated anthrax lethal toxin-induced cardiac contractile and intracellular Ca^2+ ^anomalies. The catalase-offered beneficial effects against lethal toxin were closely associated with the alleviation of lethal toxin-induced accumulation of O_2_^- ^and ROS, loss of mitochondrial membrane potential, increase in ubiquitination, decrease in proteasome activity and induction of autophagy, depicting a possible role autophagy and mitochondrial integrity in catalase-offered protection against lethal toxin. Our data further revealed that autophagy induction mitigated catalase-offered a cardioprotective effect, consistent with the finding of obliteration of lethal toxin-induced cardiomyocyte contractile anomalies. These findings collectively support a causal role of autophagy in lethal toxin-induced cardiac injury and catalase enzyme-offered cardioprotection.

Ample experimental evidence has revealed hemodynamic and cardiac anomalies following anthrax exposure [1,13-15,17,33,34]. Data from our current study revealed that anthrax lethal toxin inhibits cardiomyocyte contractile function and intracellular Ca^2+ ^handling including depressed peak shortening amplitude and maximal velocity of shortening/re-lengthening, prolonged duration of re-lengthening as well as reduced electrically-stimulated intracellular Ca^2+ ^rise (ΔFFI), and delayed intracellular Ca^2+ ^clearance. These results are consistent with our previous report [14]. Intriguingly, lethal toxin-induced anomalies in cardiac contractile and intracellular Ca^2+ ^properties were significantly attenuated or mitigated by overexpression of the antioxidant catalase. These findings denote a possible role of intracellular Ca^2+ ^homeostasis in lethal toxin- and/or catalase-elicited mechanical responses. Our observations of unchanged intracellular Ca^2+ ^regulatory proteins, including SERCA2a, Na^+^-Ca^2+ ^exchanger and phospholamban, were consistent with our earlier report [14], suggesting possible involvement of certain post-translational modification process in these intracellular Ca^2+ ^regulatory proteins en route to altered intracellular Ca^2+ ^homeostasis. In our hands, catalase overexpression alleviated lethal toxin-induced oxidative stress (O_2_^- ^and ROS) and autophagy, favoring a possible role of oxidative modification and autophagy regulation in intracellular Ca^2+ ^homeostasis in our experimental setting.

Autophagy is a tightly regulated cellular process through which mammalian cells degrade and recycle protein aggregates and organelles [18]. Under physiological conditions, autophagy helps to maintain the amino acid pool during starvation, and prevents neurodegeneration, aging and tumor development through clearance of intracellular microbes [18,35,36]. Impaired autophagy is often associated with cardiac diseases due to poor autophagic removal of damaged cellular components [35]. Autophagy can be activated by pathophysiological stress stimuli such as, hypoxia, energy depletion and ER stress as well as bacterial, viral and parasite infections [35,36]. Although up-regulated autophagy serves to offset cardiac hypertrophy via protein degradation [37,38], excessive autophagy usually compromises cardiomyocyte survival and subsequently ventricular function. Enhanced autophagy is observed in failing hearts and a wide array of cardiovascular diseases resulting in cardiac death and impaired cardiac performance [39-41]. Data from our study revealed that lethal toxin exposure induced overt myocardial autophagy, the effect of which was mitigated by catalase overexpression and NAC. More importantly, our data revealed that induction of autophagy with rapamycin nullified the cardioprotective benefit of catalase overexpression against lethal toxin whereas autophagy inhibition using 3-MA mimicked catalase-elicited beneficial effect. These data convincingly support a causality relationship of autophagy in lethal toxin- and catalase enzyme-induced cardiac mechanical responses. The fact that NAC ablated lethal toxin-induced up-regulated in autophagy (Figure 8A-D) further depicts a permissive role of oxidative stress in lethal toxin-induced induction of autophagy. ROS has been shown to activate starvation-induced autophagy, antibacterial autophagy and autophagic cell death through distinct mechanisms, depending on cell types and stimulation conditions [42]. It is unclear at this time why NAC treatment itself lowered Beclin-1 levels, which may be related to intercellular redox state-governed autophagy initiation.

Mitochondrion plays a pivotal role in energy generation, intracellular Ca^2+ ^homeostasis and ROS production [43,44]. In addition to its primary function for energy generation to meet the high demand of the beating heart, mitochondria also regulates cell death in response to a wide variety of stress signals, such as oxidative stress, infection and DNA damage [45]. Anthrax lethal toxin stimulates ROS production in macrophages and cardiomyocytes [14]. It was also shown that mitochondrial impairment is a critical event leading to macrophage cytolysis during anthrax toxin exposure [46]. This is also supported by our early finding of the involvement of NADPH oxidase, a mitochondrial-based O_2_^-^-generating enzyme, in lethal toxin-induced cardiac anomalies [14]. Data from our study revealed that anthrax lethal toxin exposure stimulates O_2_^- ^and ROS generation and reduces mitochondrial membrane potential. Interestingly, lethal toxin-induced changes in O_2_^-^, ROS and mitochondrial membrane potential were attenuated or ablated by catalase overexpression, suggesting a likely role of mitochondria in catalase-offered protection against lethal toxin exposure. Nonetheless, it is surprising for the catalase enzyme to attenuate the catalase-resistant O_2_^- ^generation. Although the precise mechanism responsible for such unexpected inhibition is still elusive at this time, a couple of scenarios may be considered. It is possible that catalase enzyme may rapidly clear the H_2_O_2 _produced by SOD, thus favoring a rightward shift for superoxide dismutation reaction to facilitate O_2_^- ^removal. In addition, the appearance of O_2_^- ^in myocytes could be induced by the external H_2_O_2 _[47]. It is plausible to speculate that catalase-induced partial attenuation of O_2_^- ^production may be associated with the reduction of H_2_O_2 _levels. Our data also suggested that damaged mitochondria in response to a lethal toxin challenge may be related to induction of autophagy. Although efficient removal of dysfunctional mitochondria by way of autophagy is critical for the maintenance of cell homeostasis, excessive autophagy induction, (in particular, mitophagy), may trigger loss of ATP production and mitochondrial membrane potential leading to mitochondrial injury [48,49]. Further study is warranted to examine the putative mechanisms whereby alterations in the autophagic removal of damaged mitochondria intervene in the process of lethal toxin toxicity.

Ubiquitin proteasome system (UPS) plays an essential role in regulating a wide variety of cellular pathways, including cell growth and proliferation, apoptosis, protein quality control DNA repair, transcription and immune response [50-52]. UPS degrades both mis-folded and damaged proteins by covalently attaching ubiquitin to target proteins followed by proteasomal degradation of these proteins. Nonetheless, proteasome functional insufficiency (PFI) often develops under pathophysiological conditions as a result of impaired proteasome activity or insufficient activity to cope with the increased demand. Proteasome functional insufficiency may lead to decreased degradation of mis-folded proteins, resulting in accumulation of protein aggregates to further inhibit proteasomal activities and deteriorate cellular stress. Increased protein ubiquitination and aberrant protein aggregation are typical signs of PFI, which is commonly seen in cardiac proteinopathy, myocardial ischemia-reperfusion injury, idiopathic dilated cardiomyopathy and hypertrophic cardiomyopathy [53-55]. PFI may compromise cardiac function through impairing protein quality control [56]. Data from our study revealed a significant increase in ubiquitination accompanied by impaired proteasome activity in response to lethal toxin challenge. Our data also revealed a reminiscent inhibitory effect of H_2_O_2 _on proteasomal activity, the effect of which was reversed by the antioxidant NAC. One likely explanation for the decreased proteasome activity following lethal toxin exposure could be due to the inactivation of 26S proteasome subunits by oxidative modification [57]. We and others have reported overt O_2_^- ^accumulation in neutrophils, macrophages and myocardium following lethal toxin exposure [14,20,21]. Inhibition of 26S proteasome could be caused by oxidative products, such as protein aggregates, oxidized lipids or by oxidative modification (4-hydroxy-nonenalyation) of several proteasome subunits [35,42,52]. Moreover, oxidative stress can induce the dissociation of the 20S core particle from the 19S regulatory particle of the 26S proteasome, which results in loss of the activities of the 26S proteasome and thus results in accumulation of ubiquitinated proteins [22]. Cardiac-specific catalase overexpression effectively mitigates lethal toxin-induced impairment of ubiquitin proteasome function (although not in ubiquitin level), suggesting a possible role of UPS in the antioxidant-offered beneficial effect against lethal toxin.

## Conclusions

Data from our study revealed that the catalase overexpression rendered cardioprotection against anthrax lethal toxin. Cardiac catalase overexpression rescues cardiac contractile dysfunction and intracellular Ca^2+ ^mishandling in response to lethal toxin exposure, possibly through alleviation of oxidative stress, mitochondrial damage and autophagy as well as improved UPS proteasomal activity. These findings have depicted potential therapeutic promises for molecules capable of scavenging ROS and suppressing autophagy in the clinical management of anthrax infection-induced cardiovascular complications.

## Abbreviations

± dL/dt: Maximal velocity of shortening/re-lengthening; 3-MA: 3-methyladenine; CM-H2DCFDA: Fluoroprobe 5-(6)-chloromethyl-2, 7-dichlorodihydrofluorescein diacetate; DHE: dihydroethidium; ER: Endoplasmic reticulum; GFP: Green fluorescence protein; LeTx: Anthrax Lethal Toxin; NAC: N-acetyl-cysteine; PLB, Phospholamban; PS: Peak shortening; ROS: Reactive oxygen species; SERCA: Sarco(endo)plasmic reticulum Ca^2+^-ATPase; TPS: Time-to-90% peak shortening; TR_90_: Time-to-90% re-lengthening; UPS: Ubiquitin Proteasome System.

## Competing interests

The authors declare that they have no competing interests.

## Authors' contributions

MRK participated in the study design, carried out *in vivo *studies, acquired contractile and Western blot data, performed *in vitro *experiments, performed statistical analysis and drafted the manuscript. XY participated in Western blot experiments. AEF participated in the design, coordination and manuscript writing. JR participated in the study design, performed statistical analysis and took part in manuscript writing. All authors have read and approved the final version of the manuscript.

## Pre-publication history

The pre-publication history for this paper can be accessed here:

http://www.biomedcentral.com/1741-7015/10/134/prepub

## Supplementary Material

Additional file 1 Figure S1: Effect of catalase overexpression on LeTx exposure-induced changes in intracellular Ca^2+ ^regulatory proteins. **A**: Representative gel blots depicting expression of SERCA2a, Na^+^-Ca^2+ ^exchanger (NCX), phospholamban (PLB), phosphorylated PLB and GAPDH (used as loading control); **B**: SERCA2a; C: NCX; and D: phosphorylated-PLB (p-PLB)-to-PLB ratio. Mean ± SEM, n = 6 mice per group, *P <0.05 *vs*. WT group. Figure S2: Effect of autophagy induction or inhibition on lethal toxin induced cardiac contractile dysfunction. **A-B**: Isolated cardiomyocytes from WT and CAT mice were incubated with lethal toxin (100 ng/ml) for 3 h in presence or absence of the autophagy inhibitor 3-methyladenine (3-MA, 10 mM) or autophagy inducer rapamycin (5 μM) respectively. A: Resting cell length; B: time-to-peak shortening (TPS). Mean ± SEM, n = 60 to 75 cells from three mice per group, *P <0.05 *vs*. WT group, # *P *<0.05 *vs*. WT-LeTx group. Click here for file
