# Supplementary material for: Cardiac-specific catalase overexpression rescues anthrax lethal toxin-induced cardiac contractile dysfunction: role of oxidative stress and autophagy
Source: BMC Med. 2012 Nov 7;10:134. doi: 10.1186/1741-7015-10-134 (PMC3520786; doi:10.1186/1741-7015-10-134)
Supplement: Additional file 1 — Figure S1: Effect of catalase overexpression on LeTx exposure-induced changes in intracellular Ca2+ regulatory proteins. A: Representative gel blots depicting expression of SERCA2a, Na+-Ca2+ exchanger (NCX), phospholamban (PLB), phosphorylated PLB and GAPDH (used as loading control); B: SERCA2a; C: NCX; and D: phosphorylated-PLB (p-PLB)-to-PLB ratio. Mean ± SEM, n = 6 mice per group, *P <0.05 vs. WT group. Figure S2: Effect of autophagy induction or inhibition on lethal toxin induced cardiac contractile dysfunction. A-B: Isolated cardiomyocytes from WT and CAT mice were incubated with lethal toxin (100 ng/ml) for 3 h in presence or absence of the autophagy inhibitor 3-methyladenine (3-MA, 10 mM) or autophagy inducer rapamycin (5 μM) respectively. A: Resting cell length; B: time-to-peak shortening (TPS). Mean ± SEM, n = 60 to 75 cells from three mice per group, *P <0.05 vs. WT group, # P <0.05 vs. WT-LeTx group. [file 1741-7015-10-134-S1.DOC]

**Cardiac-Specific Catalase Overexpression Rescues Anthrax Lethal Toxin-Induced Cardiac Contractile Dysfunction: Role of Oxidative Stress and Autophagy**

**Supplementary Data**

**B.**

**D.**

**C.**

**A.**

Additional file 1 Figure S1

**A.**

**B.**

Additional file 1 Figure S2

Additional file 1Figure S2
